# Whole-Exome Sequencing Implicates Neuronal Calcium Channel with Familial Atrial Fibrillation

**DOI:** 10.3389/fgene.2022.806429

**Published:** 2022-01-28

**Authors:** Oliver Bundgaard Vad, Yannan Yan, Federico Denti, Gustav Ahlberg, Lena Refsgaard, Sofia Hammami Bomholtz, Joana Larupa Santos, Simon Rasmussen, Stig Haunsø, Jesper Hastrup Svendsen, Ingrid Elizabeth Christophersen, Nicole Schmitt, Morten Salling Olesen, Bo Hjorth Bentzen

**Affiliations:** ^1^ Department of Biomedical Sciences, Faculty of Health and Medical Sciences, University of Copenhagen, Copenhagen, Denmark; ^2^ Laboratory for Molecular Cardiology, Department of Cardiology, Centre for Cardiac, Vascular-, Pulmonary and Infectious Diseases, Righospitalet, Copenhagen University Hospital, Copenhagen, Denmark; ^3^ Disease Systems Biology Program, University of Copenhagen, Copenhagen, Denmark; ^4^ Department of Clinical Medicine, Faculty of Health and Medical Sciences, University of Copenhagen, Copenhagen, Denmark; ^5^ The Department of Medical Genetics, Oslo University Hospital, Oslo, Norway; ^6^ Department of Medical Research, Bærum Hospital, Vestre Viken Hospital Trust, Rud, Norway

**Keywords:** genetics, atrial fibrillation, ion channels, cardiology, mechanisms of arrhythmia, arrhythmias (cardiac)

## Abstract

**Background:** Atrial Fibrillation (AF) is the most prevalent sustained cardiac arrhythmia, responsible for considerable morbidity and mortality. The heterogenic and complex pathogenesis of AF remains poorly understood, which contributes to the current limitation in effective treatments. We aimed to identify rare genetic variants associated with AF in patients with familial AF.

**Methods and results:** We performed whole exome sequencing in a large family with familial AF and identified a rare variant in the gene *CACNA1A* c.5053G > A which co-segregated with AF. The gene encodes for the protein variants Ca_V_2.1-V1686M, and is important in neuronal function. Functional characterization of the CACNA1A, using patch-clamp recordings on transiently transfected mammalian cells, revealed a modest loss-of-function of Ca_V_2.1-V1686M.

**Conclusion:** We identified a rare loss-of-function variant associated with AF in a gene previously linked with neuronal function. The results allude to a novel link between dysfunction of an ion channel previously associated with neuronal functions and increased risk of developing AF.

## Introduction

Atrial fibrillation (AF) is a supraventricular arrhythmia associated with increased morbidity and mortality, mainly through the increased risk of stroke and heart failure. As the most common sustained arrhythmia, the disease affects more than 30 million individuals worldwide, and with an increasing prevalence with age, this number is expected to increase significantly in the coming decades (Hindricks et al., 2021).

Numerous genes have been implicated with AF (Olesen et al., 2014), among those cardiac ion channels and proteins interacting with ion channels. Recently variants in genes encoding cytoskeletal proteins, such as *TTN* and *MYL4* have also been associated with early-onset AF (Orr et al., 2016; Gudbjartsson et al., 2017; Ahlberg et al., 2018; Choi et al., 2018). In total, large genome wide association studies (GWAS) have so far identified 134 independent genetic loci associated with AF (Nielsen et al., 2018; Roselli et al., 2018), many of which are located near genes that are important for electro-physiological function (Roselli et al., 2018). However, the GWAS loci identified so far only partially explain the heritability of AF and our knowledge of the underlying mechanisms still remain incomplete.

We hypothesized that studying familial AF occurring without evidence of other cardiovascular or pulmonary diseases could give additional insight to the complex pathophysiology of the disease, since these patients are likely to have a substantial genetic component. Therefore, we performed whole exome sequencing (WES) in patients with familial AF and identified a rare genetic variant which co-segregated with AF, in a gene previously linked to neuronal function. Subsequent functional characterization of the identified variant revealed that it resulted in dysfunctional ion channels.

Interestingly, many of the AF-associated genes identified through GWAS are more abundantly expressed in the nervous system than in the heart (*AKAP5*, *AKAP6*, *CHRNB2*, *DRD5*, *KCND3*, *NTSR1*, *PTK2*, *KCNN2* and *KCNN3*) (Uhlén et al., 2015; The Human Protein Atlas, 2021). This suggests that the nervous system could potentially play a role in the pathogenesis of AF.

## Methods

### Study Population

The study complies with the Declaration of Helsinki. Written informed consent was obtained from all participants and the study was approved by the scientific ethics committee for the Capital Region of Denmark (protocol number H-1-2011-044).

**FIGURE 1:**
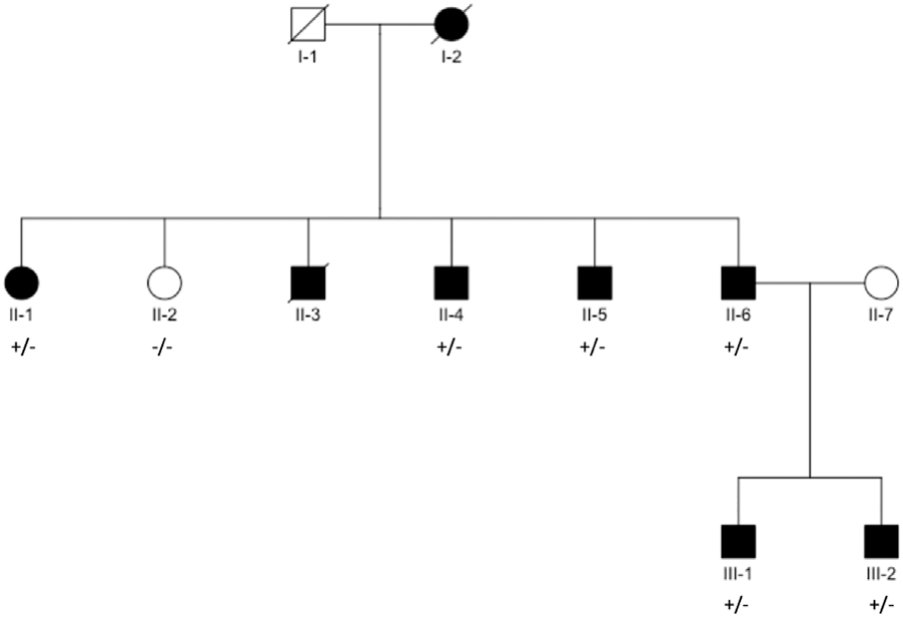
Pedigree structure. Black filled symbols indicate individuals diagnosed with AF. Square/Circle shows Male/Female. Diagonal line denotes diseased individual. Presence of variant, named in title of each panel, is indicated with “+” for presence and “–” for absence (individuals with available exomes).

We recruited seven family members from a Danish family with aggregation of AF (Figure 1); of which four were affected and one unaffected in generation II, and two were affected in generation III. AF diagnosis was defined by ICD-8 code 427.93, 427.94, and ICD-10 code I48.

### Whole Exome Sequencing

Genomic DNA was extracted from peripheral blood using a QIAamp DNA Blood Mini kit. Genomic DNA (3 μg) was randomly fragmented by Covaris, and ligated with adaptors. After purification using Agencourt AMPure SPRI beads, the adaptor-ligated templates were amplified by ligation-mediated PCR and hybridized to the SureSelect Biotinylated RNA Library for enrichment. Whole Exome sequencing (WES) was performed using the Agilent SureSelect Human All Exon Capture kit and 90-bp paired-end sequencing on an Illumina Genome Analyzer II system. For verification of variants, Sanger sequencing was conducted as previously described (Olesen et al., 2012).

We trimmed raw reads and filtered low-quality reads using cutadapt vX (Martin, 2011) and prinseq vX (Schmieder and Edwards, 2011). Alignment [with Burrow-Wheelers Aligner mem algorithm (Li and Durbin, 2009)] to the human reference genome (NCBI Build 37) and post-processing was made according to Genome Analysis Toolkit version 3.4 (GATK) guidelines (Auwera et al., 2013). Variant calling was performed with Haplotypecaller/GATK v3.4, followed by quality control and variant filtering (Supplementary Figure S1). Relatedness was inferred using the King-robust algorithm (Manichaikul et al., 2010).

### Family-Based Genetic Analysis

After bioinformatics processing of WES data, we focused on rare deleterious variants that co-segregated with AF within the pedigree. To estimate minor allele frequency (MAF) for variants, we screened the publicly available resources dbSNP and GnomAD and an in-house Danish population of 1,972 exomes (D2K) (Sherry et al., 2001; Lohmueller et al., 2013; The 1000 Genomes Project Consortium et al., 2015; Karczewski et al., 2020). A rare variant was defined as MAF<0.01% in GnomAD (in all sub-populations) or MAF<1/(2x1972) in D2K, and listed as “non-common” (or absent) in dbSNP b.153.

We screened for variants that co-segregated in all affected family members. Putative variants were ranked and prioritized using combined annotation depletion score (CADD score), a model which estimates variant deleteriousness by incorporating numerous different annotation methods, including conservation based annotations like GERP and protein-level scores like SIFT (Kircher et al., 2014). Additionally we considered the given gene’s constraint metrics, Z-score and pLi, which indicate intolerance of deleterious variation in a given gene (Lek et al., 2016).

### Expression Plasmids and Site Directed Mutagenesis

Human Ca_V_2.1 in pcDNA3.1 was a kind gift by Dr. Geoffrey Pitt, Duke University, United States.

The point mutation c.5056G > A (p.V1686M) in Cav2.1. isoform 3 (NM_001127221) was generated by GenScript, NJ, United States and verified by sequencing by GenScipt, Nj, United States.

### Cell Cultures and Patch-Clamp Electrophysiology

For Ca_V_2.1, HEK293 cells were transfected with 0.5 µg Ca_V_2.1-WT or 0.5 µg Ca_V_2.1-V1686M and co-transfected with 0.5 µg Ca_V_β2, 0.5 µg Ca_V_α2δ1 and 0.2 µg EGFP. All transfections were done using siLentFect™ Lipid (Bio-Rad, Copenhagen, Denmark) according to manufacturer’s instructions. Patch-clamp experiments were performed at room temperature. Currents were measured 72 h after transfection from single fluorescent CHO or HEK cells using a MultiClamp 700B amplifier and MultiClamp Commander (Molecular Devices, Axon Instruments, Sunnyvale, California, United States). The cells were superfused with an extracellular solution containing the following (in mM): 140 TEA-Cl, 3 CsCl, 2.5 CaCl_2_, 1.2 MgCl_2_, 10 HEPES and 10 Glucose, pH adjusted to 7.4 with NaOH. Pipettes were pulled from borosilicate glass capillaries (Harvard Apparatus, Holliston, United States) using a DMZ Universal Puller (Zeitz Instruments, Martinsried, Germany) and had a resistance of 4.0-6.0 MΩ when filled with intracellular solution containing the following (in mmol/L): 140 CsCl, 1 EGTA, 4 Na_2_ATP, 0.1 Na_3_GTP and 10 HEPES, pH adjusted to 7.2 with CsOH. Data were acquired using a Digidata 1,440 Converter and the software pClamp 10.4 Commander (Molecular Devices).

### Voltage Clamp Protocols and Patch Clamp Data Analysis

Ca_V_2.1 currents were elicited by applying 20 ms voltage steps from −60 to 70 mV in 5 mV steps, from a holding potential of −80 mV. The measured peak current at the beginning of each voltage step was normalized to the cell capacitance and plotted as a function of the test potential in order to generate current-voltage relationships. The voltage dependence of activation was measured by calculating the reversal potential (V_rev_) of each experiment by measuring the linear regression of the curve between 10 and 30 mV and using it to calculate the conductance (G) using formula G = I/(V_m_-V_rev_), where I is the current and V_m_ the membrane potential. We then normalized each experiment for the maximum G. To study the voltage dependence of inactivation, we applied a 30 s ladder of 5 mV voltage steps from -100 mV to +30 mV and subsequently measured the peak Ca^2+^ current at 20 mV. We then normalized to maximal Ca^2+^ current and determined the V_50_ of inactivation. The time constant (τ) of activation was measured by fitting single exponential functions to the activation phase of the Ca^2+^ current. The τ of deactivation was calculated by fitting single exponential functions to the tail currents obtained by first eliciting the current to 20 mV for 20 ms, followed by 30 ms test pulses between −80 and 10 mV, in 5 mV steps. The τ of inactivation was measured by fitting a single exponential function to the inactivation phase of the current during a 3 s voltage step from a holding of −80–20 mV. Statistical analysis of the current-voltage relationship, the activation and the deactivation time constants were performed with 2-way ANOVA, with a Sidak’s multiple comparison post-test. Statistical analysis of V_50_ of inactivation and activation were done with Student’s t-test; *p* < 0.05 was considered statistically significant.

## Results

### Clinical Characteristics

Eight family members across three generations were affected by AF in the family (Figure 1). Of the affected family members, seven participated in the study and were available for sequencing, while one was deceased at the time of inclusion. Additionally, we included one final family member who had not been diagnosed with AF.

The proband, III-1, had onset of AF at age 37. He had no known risk factors for AF other than obesity [Body Mass Index (BMI) = 37.8] and an echocardiography with a marginally enlarged left atrium. His only reported symptom was dyspnea during physical activity. His brother, III-2, was diagnosed with persistent lone AF at the age of 38. His only reported symptom was fatigue. Electrocardiogram (ECG) and echocardiography were normal, without signs of structural heart disease. He went through electrical cardioversion several times within the first 6 months, but due to recurrence of AF, treatment with Flecainide was initiated. Fourteen days after initiation of Flecainide treatment he went through a witnessed cardiac arrest (SCD) and was resuscitated within 11 min. Brugada syndrome was suggested and a genetic variant in *SCN5A* was found (rs41311117, ENST00000333535, p.F2004L). However, subsequent ECG, echocardiography and coronary angiography were all normal, and the suggested diagnosis of Brugada syndrome was rejected.

Their father, II-6, had onset of AF at the age of 40. Due to difficulty controlling his AF, he was treated with a His-bundle ablation and a pacemaker. In the second generation of the pedigree, four of five were diagnosed with early-onset AF. Sibling II-2 was not diagnosed with AF, despite several predisposing risk factors (BMI = 34.4, hypertension and diabetes).

Following identification of the variant in *CACNA1A*, follow-up interviews were conducted with five of the six family members to uncover potential neurological symptoms. Proband III-1 reported symptoms of intense, monthly headaches with nausea and increased light sensitivity compatible with migraine. These symptoms disappeared after the patient started treatment with metoprolol, a β_1_-receptor blocker, for his AF. Of five family members, three reported restless legs, four reported muscle cramps, and three reported problems with loss of balance. Clinical characteristics and neurological symptoms have been reported in Table 1 and Table 2 respectively.

**TABLE 1:** Clinical characteristics of family members.

ID	Sex	AF onset (years)	AF type	Symptoms	Comorbidities before AF diagnosis or within 1 year after	Diagnostic Procedures	Notes
II-1	F	57	Perm	Palpitations	none	Echo: Normal	—
II-2	F	NA	NA	NA	HCL, obesity, HT, Diabetes	NA	No AF.
II-4	M	48	Perm	Dyspnea	HCL	Echo: Arrhythmia induceret cardiomyopathy. Remission with control of AF.	—
II-5	M	47	Perm	Unknown	None	Echo: Moderate pulmonary hypertension	—
II-6	M	40	Perm	Presyncope, syncope	HT	Echo: Normal, CAG: Normal	Difficulty to control AF - > His bundle ablation and pacemaker
III-1	M	37	Perm	Dyspnea with activity	obesity	Echo: Marginally enlarged left atrium (73 ml)	—
III-2	M	38	Per	Fatigue, dyspnea	none	Echo: Normal, CAG: Normal	Aborted SCD. ICD
						Flecanaid test: Negative, No sign of Brugada	

HCL, Hypercholesteremia; HT, Hypertension; HF , Heart Failure; SCD, Sudden Cardiac Death; ICD, Implanted Cardioverter Defibrillator. Echo = Echocardiogram. CAG, Coronary Angiogram. M/F = Male/Female. Per. = Persistent. Perm = Permanent.

**TABLE 2:** Neurological symptoms in CACNA1A-V1686M carriers.

ID	Sex	AF type	AF onset (years)	Gene	Frequent or intense head-aches	Light/sound sensitivity during headaches	Nausea during head-aches	Symptoms of ataxia	Rest-less legs	Muscle cramps	Family history of migraine	Comment:
II-1	F	Perm	57	*CACNA1A*	No	No	No	Loss of balance^a^	Yes	Yes	Yes	Underdeveloped lower extremities due to accident as a child Balance problems appear and disappear suddenly
II-4	M	Perm	48	*CACNA1A*	No	No	No	None	Yes	Yes	Yes	No
II-5	M	Perm	47	*CACNA1A*	No	No	No	Loss of balance^a^	No	Yes	Yes	Sudden development of balance problems 1 year ago Grandchild with migraine
II-6	M	Perm	40	*CACNA1A*	No	No	No	Yes, loss of balance and loss of fine motor control^a^	Yes	Yes	Yes	Problems with balance and fine motor skills after injury to back
III-1	M	Perm	37	*CACNA1A*	Yes^a^	Yes^a^	Yes^a^	None	No	No	Yes	Frequent/intense headaches, including nausea and light sensitivity, occurred monthly before beta-antagonist treatment
III-2	M	Per	38	*CACNA1A*	NA	NA	NA	NA	NA	NA	Yes	Did not wish to participate in follow-up interview

a See comment. AF, Atrial Fibrillation. M/F = Male/Female. Per. = Persistent. Perm = Permanent.

### Sequencing Quality and Relatedness

Sequencing coverage for the family was determined in the targeted region with a padded region of 100 base pairs (bp) on each side. The mean coverage in this region was 85X (low-high = 50X-96X; Supplementary Table S1). In the region, 88.7% (low-high = 85.0-90.3%) of bases were covered >10X and 79.8% (low-high = 73.7-82.0%) >20X. The mean transition transversion ratio (TiTv) was 2.72 (low-high = 2.67-2.78; Supplementary Table S1).

The relatedness analyses, using KING robust algorithm, agreed with reported relatedness and pedigree structure (Figure 1, Supplementary Figure S2, Supplementary Table S2). Variants were verified using Sanger Sequencing in all participants (Supplementary Figure S3).

### Genetic Variation

Three putative variants met the criteria in the family (Table 3). Based on CADD score, the top ranked variant was the *CACNA1A* variant (ENST00000573710). Interestingly, the variant resides in the top 99th percentile of the most constrained coding regions (Havrilla et al., 2019). The *CACNA1A* gene encodes the alpha subunit Ca_V_2.1 of the P/Q type calcium channel. Rare variants in proximity to V1686M in Ca_V_2.1 have previously been associated with episodic ataxia type 2, familial hemiplegic migraine and spinocerebellar ataxia type 6, demonstrating the importance of the P/Q type current for the nervous system (Ducros et al., 2001).

**TABLE 3:** Rare co-segregating variants in family members.

Gene	Genomic postion	RefSNP	Transcript	AA change	Type	Consequence	MAF		Prediction		
							GnomAD	D2K	CADD	SIFT	GERP
CACNA1A	19:13346442C > T	NA	ENST00000360228	p.V1686M	SNV	Missense	0	0	28	D	5.0
CD163L1	12:7521563TGA > T	rs781351459	ENST00000539726	S1RfsX64	DEL	Frameshift	0.3526	0	7.034	NA	NA
OR8U1	11:56143415A > G	NA	ENST00000302270	p.T106A	SNV	Missense	0	0	0.001	T	−7.1

RefSNP, Reference single-nucleotide polymorphism; AA, Amino acid; SNV, Singe nucleotide variant; DEL, Deletion; MAF, Minor allele frequency. GnomAD, Genome Aggregation Database. D2K = 2000 Danish Exomes. CADD, Combined Annotation Dependent Depletion(15). SIFT, Sorting Intolerant from Tolerant(16). D = Deleterious. T = Tolerated. GERP, Genomic Evolutionary Rate Profiling Score (17).

All variants were confirmed by Sanger sequencing. We performed whole exome sequencing on seven individuals in total from the family.

### Functional Characterization of Identified Rare *CACNA1A* Variant

#### Ca_V_2.1-V1686M Shifts the Voltage Dependence of Activation to more Depolarized Potentials and Accelerates the Inactivation.

**FIGURE 2:**
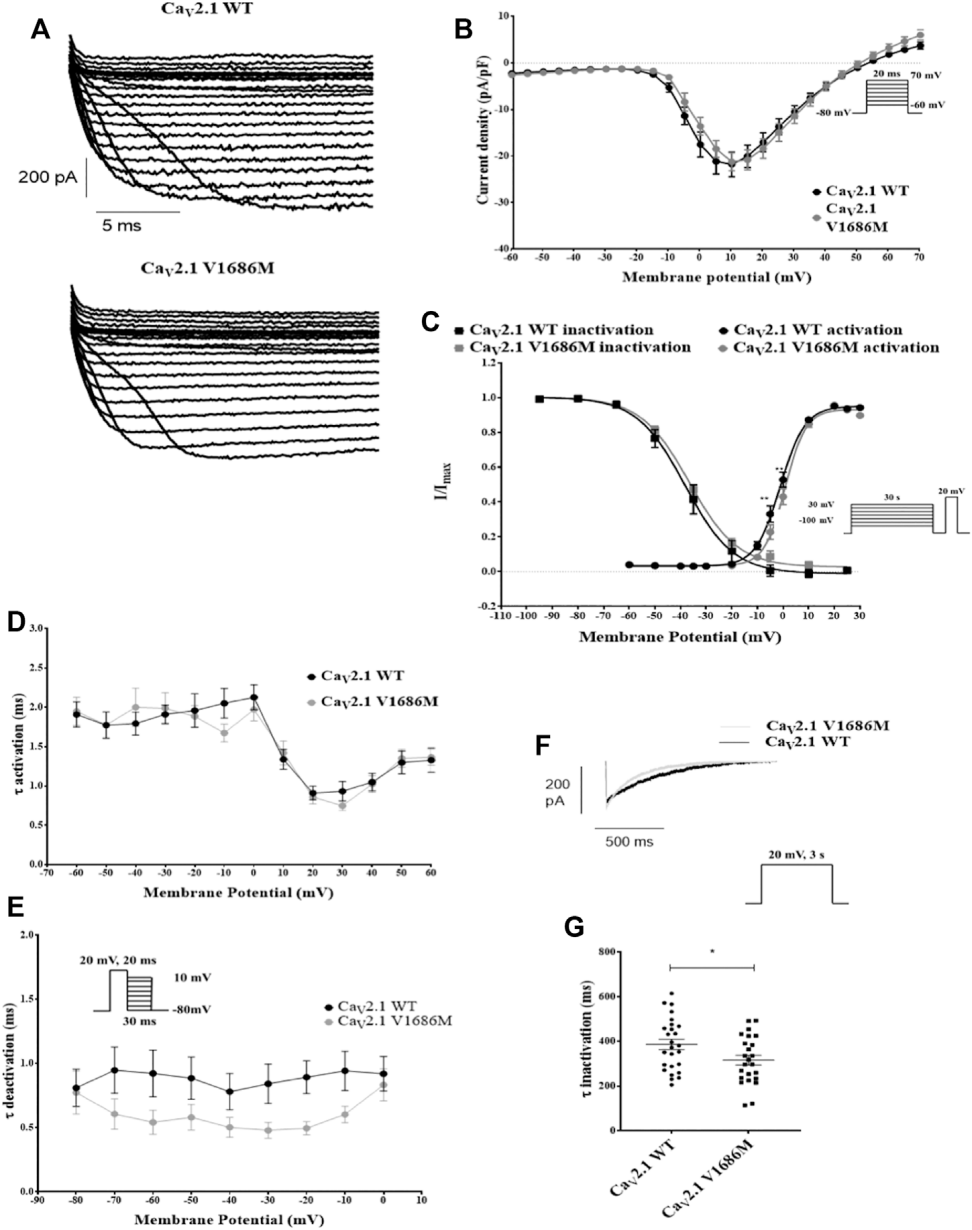
Functional characterization of CaV2.1-V1686M. **(A)**: Representative traces for currents elicited in transiently transfected HEK293 cells for Ca_V_2.1-WT and Ca_V_2.1-V1686M. **(B)**: Current/voltage relationship of the peak current density for Ca_V_2.1-WT (black, n = 32) and Ca_V_2.1-V1686M (grey, n = 25). The voltage protocol is shown in the inset. **(C)**: Steady-state activation and inactivation curves for Ca_V_2.1-WT (black, activation n = 30, inactivation n = 20) and Ca_V_2.1-V1686M (grey, activation n = 20, inactivation n = 18). The voltage protocol is shown in the inset. **(D)**: Activation time constants for Ca_V_2.1-WT (black) and Ca_V_2.1-V1686M (grey). **(E)**: Deactivation time constants for Ca_V_2.1-WT (black) and Ca_V_2.1-V1686M (grey). **(F)**: Representative current trace for Ca_V_2.1-WT (black) and Ca_V_2.1-V1686M (grey) elicited by the voltage protocol shown in the inset, demonstrating slow inactivation. **(G)**: Time constant for the slow inactivation Ca_V_2.1-WT (black, n = 26) and Ca_V_2.1-V1686M (grey, n = 24). For figure B–E the statistical analysis was performed with 2-way ANOVA, followed by a Sidak’s multiple comparison post-test; For figure G a unpaired student’s t-test was performed. *p* < 0.05 was considered statistically significant.

To investigate the effect of the variant V1686M we transiently transfected HEK293 cells with Ca_V_2.1 wild-type (WT) or Ca_V_2.1-V1686M. We co-transfected with Ca_V_2.1 ancillary subunits Ca_V_β2 and Ca_V_α2δ1 (Figure 2). As expected, in cells expressing the WT channel membrane depolarization gave rise to a fast activating and slowly inactivating current. Mutant Ca_V_2.1 also produced inward calcium currents with similar peak current density for Ca_V_2.1-V1686M as compared to WT (-21.2 ± 1.9 pA/pF vs -21.8 ± 2.6 pA/pF; *p* = 0.99) (Figure 2A,B and Supplementary Table S4). However, we observed a small but significant depolarizing shift of 3.5 mV in the voltage dependence of activation for V1686M compared to WT (2.4 ± 0.6 mV vs -1.0 ± 0.8 mV; *p* = 0.0034) (Figure 2C and Supplementary Table S4), but there was no difference in the time constant of activation or deactivation (Figures 2D,E). We also addressed whether the mutation affected the inactivation of the channel. When comparing the voltage dependence of steady-state inactivation, we observed no changes (WT vs Ca_V_2.1-V1686M: 39.0 ± 2.7 mV vs -35.7 ± 1.3 mV; *p* = 0.2326); however, the time constant of inactivation was significantly reduced from 386 ± 23 ms (WT Ca_V_2.1, n = 26) to 316 ± 22 ms (Ca_V_2.1-V1686M, n = 24) (Figures 2F,G).

#### Gene Expression Analysis

To assess the expression levels of the Ca_V_2.1 channel in the human heart, we measured mRNA levels in the left and right atrium and left and right ventricle in seven healthy individuals by quantitative PCR (Supplementary Methods). *CACNA1A* mRNA was detected in both atrial and ventricular samples (Supplementary Figure S4).

## Discussion

AF is a complex polygenetic disease, and many genetic loci across the genome have already been associated with AF (Nielsen et al., 2018; Roselli et al., 2018), however the complete genetic component of AF has yet to be uncovered. In this study we hypothesized that examining families with aggregation of AF cases could yield novel insights into the genetics of AF, as individuals in these families were likely to have a considerable genetic disposition for AF. Using this approach, we identified a novel variant in the gene *CACNA1A*, encoding the P/Q type calcium channel α-subunit Ca_V_2.1, which co-segregated with AF in a large family with an autosomal dominant inheritance pattern of AF (Figure 1). In this family, spanning three generations, eight individuals had been diagnosed with AF before the age of 60. Of the seven family members from generation II and III available for WES, the novel variant was shared by all affected individuals, whereas the unaffected sibling (II-2) did not carry the variant. Of the three co-segregating variants in the family, this variant was predicted to be the most likely to be deleterious.

Interestingly, the *CACNA1A* gene also resides in one of the most constrained coding regions of the human genome. Mutants found in the highest percentile of constrained coding regions are more likely to cause severe developmental phenotypes and are enriched for pathogenic variants in ClinVar, a public database of variant-phenotype relationships (Havrilla et al., 2019). The Ca_V_2.1 channel encoded by the *CACNA1A* gene is expressed at the pre-synaptic axon terminal. It regulates calcium entry that triggers neurotransmitter release and is an important target for control of neuronal activity (Nanou and Catterall, 2018). Functional characterization of the ion channel encoded by the variant, Ca_V_2.1-V1686M, showed a significant shift of the voltage dependence of activation towards more depolarized potentials and an increased speed of inactivation, both resulting in loss-of-function of Ca_V_2.1.

Other genetic variants in *CACNA1A* have previously been linked with various neurological disorders e.g. episodic ataxia type 2 and familial hemiplegic migraine (Jen and Wan, 2018). Interestingly, several of the family members participating in this study reported symptoms indicative of such disorders in follow-up interviews. For instance, several individuals reported periods of loss of balance, a common symptom for episodic ataxia type 2, while others reported symptoms of migraine as well as muscle cramps and restless legs (Table 2). While these neurological symptoms are self-reported responses that may be incidental, we speculate that abnormal neuronal activity in certain parts of the brain-heart axis could predispose the affected individuals in the family to AF based on the importance of these channel proteins for controlling neuronal excitability. The co-segregation of loss-of-function variants in the gene encoding Ca_V_2.1 in this family, and the low-level mRNA expression of Ca_V_2.1 identified in human atria further underline this hypothesis. Furthermore, while the functional effects of the variant were somewhat modest, previous studies have demonstrated the importance and arrhythmogenic potential of calcium channels in AF (Benzoni et al., 2020).

The involvement of regulation from the autonomic nervous system on AF pathogenesis is well established (Ripplinger et al., 2016). Interestingly, by using pharmacological blockers of P/Q-channels it has been found that Ca_V_2.1 is important for cardiac vagal excitation and for the neuronal parasympathetic activity of guinea pig atria (Hong and Chang, 1995; Wang et al., 2001). Moreover Ca_V_2.1 activity is important for heart rate control in mice as demonstrated by the lower heart rate observed in the mouse model (*rolling Nagoya* (tg^rol^)) that carries a mutation in *CACNA1A* causing reduced Ca_V_2.1 current (Ohba et al., 2009). In light of this, we speculate that this novel variant in *CACNA1A* may disturb the autonomic regulation of the heart and may act as a modifier that affects multiple genes and, depending on co-inheritance of other variants, could influence predisposition for AF. Such genetic modifiers have previously been shown to be involved in human heart disease (Gifford et al., 2019).

However, our study also has some limitations which must be addressed. First, the participating individuals were all of European ancestry. Additionally, a majority of the affected family members were male. It cannot be excluded that these factors may limit the generalizability of our results. Secondly, while whole-exome sequencing is a thorough method of investigating genetic variation, it only covers the protein coding parts of the genome, and it therefore cannot be excluded that some of the individuals may also carry variants in non-protein coding regions, that may affect AF risk. It should also be noted that while the variant was found to significantly affect electrophysiological properties, this effect was rather modest. As this was only identified in one family and the size of this family limited the opportunities for conducting additional statistical analyses, our results should be interpreted with caution. Additionally, while the CACNA1A gene was expressed in cardiac tissue, it was not determined whether the variant influenced arrhythmia through neurons or the function of cardiomyocytes. Finally, a variant in the *SCN5A* gene (rs41311117) was identified in a single family member. While this particular *SCN5A* variant has a relative high MAF (e.g. MAF in GnomAD European population 0.2%) and its pathogenicity has recently has been debated (Risgaard et al., 2013; Ghouse et al., 2017), it cannot be excluded that it may have contributed to arrhythmia in this individual.

In conclusion, our results provide new insights into the role of the autonomic nervous system in AF pathogenesis and elucidate a possible novel mechanism of neuronal function in predisposition for cardiac arrhythmia. They should however be interpreted with caution until they have been replicated in other families and larger cohorts.

## Data Availability

The datasets presented in this article are not readily available because of patient confidentiality reasons. Requests to access the datasets should be directed to the corresponding author.
