# Supplementary material for: Whole-Exome Sequencing Implicates Neuronal Calcium Channel with Familial Atrial Fibrillation
Source: Front Genet. 2022 Jan 28;13:806429. doi: 10.3389/fgene.2022.806429 (PMC8832975; doi:10.3389/fgene.2022.806429)
Supplement: Supplementary file 1 [file DataSheet1.docx]

**Supplemental material**

OB Vad, et al. **Whole-exome sequencing implicates neuronal calcium channel with familial atrial fibrillation**

Table of contents

[Supplementary Methods 3](#_Toc90926780)

[Gene expression analysis 3](#_Toc90926781)

[Supplementary Figures 5](#_Toc90926782)

[Supplementary Figure S1. Exome sequencing pipeline. 5](#_Toc90926783)

[Supplementary Figure S2. Relatedness in family members. 6](#_Toc90926784)

[Supplementary Figure S3. Sanger Sequencing Electropherogram 7](#_Toc90926785)

[Supplementary Figure S4. Expression of *CACNA1A* in cardiac chambers 8](#_Toc90926786)

[Supplementary tables 9](#_Toc90926787)

[Supplementary Table S1. Exome sequencing statistics. 9](#_Toc90926788)

[Supplementary Table S2. Reported and observed relatedness in Family A 10](#_Toc90926789)

[Supplementary Table S3. Electrophysiological parameters 11](#_Toc90926790)

[Supplementary References 12](#_Toc90926791)

# Supplementary Methods

## Gene expression analysis

Tissue biopsies from seven healthy donor hearts unsuitable for transplantation, five women (age 17-54 years) and two men (age 21 and 44 years), were provided by Dr. András Varró (University of Szegeb, Hungary) under ethical approval number 4991-0/2010-1018EKU (339/PI/010). The samples have been used previously by Skarsfeldt et al. (1). Samples from the four cardiac chambers from each patient were available. Left ventricle biopsies were separated into endo-, myo- and epicardium. The biopsies were snap-frozen in liquid nitrogen immediately after dissection and stored at -80°C until further use.

Total RNA including small RNAs was isolated from cardiac tissue biopsies. Briefly, 40 mg of tissue was homogenized in QIAzol reagent (QIAGEN, Maryland, USA) using a Precellys 24 homogenizer (Bertin Technologies, Montigny-le-Bretonneux, France). Total RNA was purified using the miRNeasy Mini kit (QIAGEN, Hilden, Germany) according to manufacturer’s instructions. DNase treatment was performed to eliminate genomic DNA contamination. The RNA purity and concentration were assessed by spectrophotometry in a NanoDrop2000 (ThermoScientific, Wilmington, USA). Polyadenylated RNAs were reverse-transcribed to cDNA using the Nanoscript2 Reverse Transcription kit (Primerdesign Ltd, Southampton, UK) according to manufacturer’s instructions.

Real-time quantitative polymerase chain reaction (qPCR) was carried out to measure the expression of *CACNA1A*. Reactions were run in triplicates using Taqman double dye probes and PrecisionPLUS MasterMix with ROX (Primerdesign Ltd, UK) on a CFX Connect Real-Time System (BIO-RAD, Hertfordshire, UK) using the following steps: 95°C for 2 min followed by 50 cycles of 95°C for 15 s and 60°C for 1 min.

The followin*g* forward and reverse primer sequences were used: GATACTTTTCCAGCAGCAATAATGA (*CACNA1A*_Forward), ACTTGATCCCGTCGTACATGA (*CACNA1A*_Reverse). The genes *YWHAZ, ACTB* and *RPL13A* were used as reference to normalize the data. Threshold cycle (C*t*) values were collected via Bio-Rad CFX96 Manager 3.0 software in the single threshold mode and transferred to a spreadsheet for calculation of ΔC*t*s. Relative expression values were obtained using the 2^-ΔCt^ method

# Supplementary Figures

### Supplementary Figure S1. Exome sequencing pipeline.

Diagram for the pipeline followed in this project for processing the sequence data. Additional samples from 1000 Genomes project, added for improvement of soft filtering, have been treated in the same way, with exception of merging of bam files before removing duplicates with Piccard’s tools. GATK, Genome Analysis Tool Kit; BQSR, Base Quality Score Recalibration; VQSR, Variant Quality Score Recalibration.


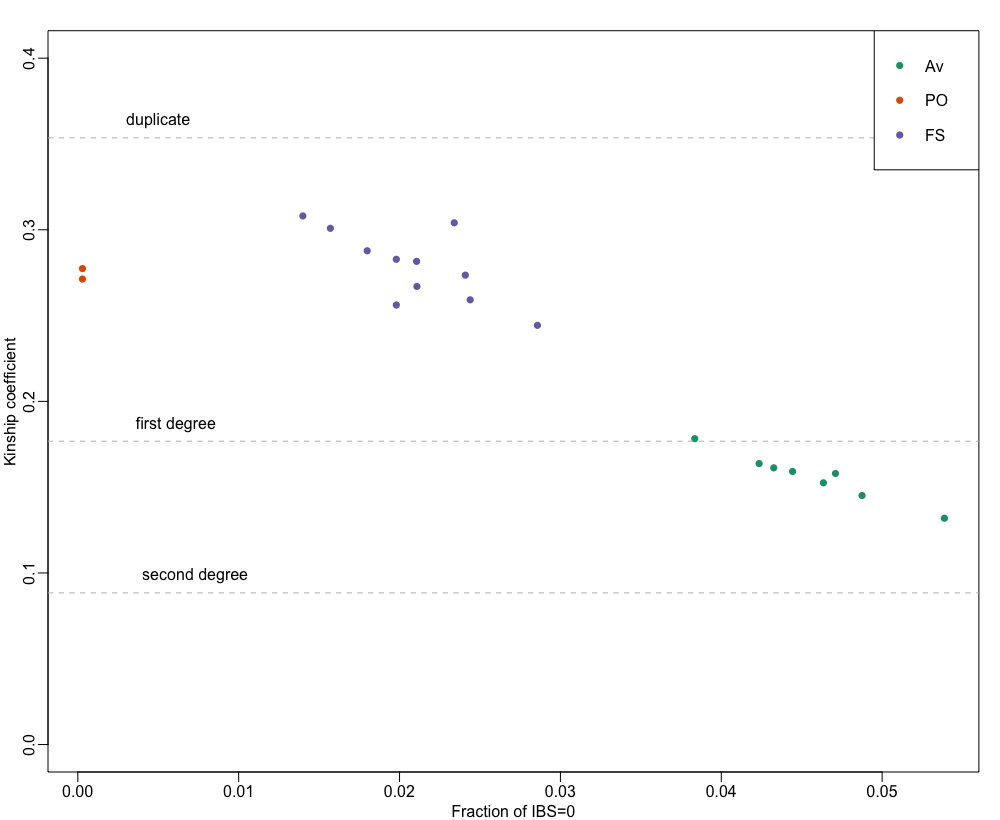


### Supplementary Figure S2. Relatedness in family members.

Inference of relatedness in Family using the KING robust algorithm with exome sequencing data. Estimated kinship coefficients are shown on the y-axis and proportion of zero identity by state (IBS) is shown on the x-axis. Top dashed line marks the threshold for duplicated or identical twin pairs. Middle and bottom dashed lines show threshold for first- and second-degree pairs, respectively. Reported relatedness are marked by color, coded in the legend (Av = Avuncular, PO = Parent offspring, FS = First sibling).


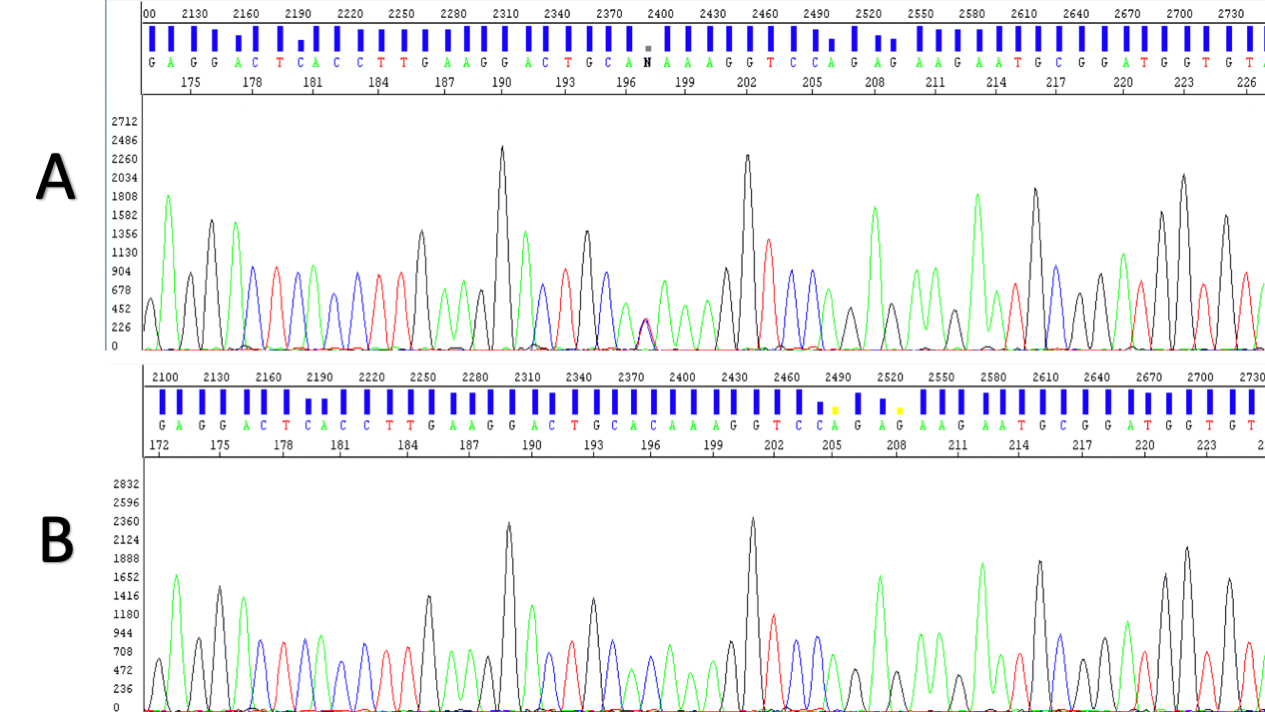


### Supplementary Figure S3. Sanger Sequencing Electropherogram

Sanger sequencing was conducted to confirm the variant in all participants. Figure S1A illustrates the presence of the *CACNA1A* c.5053G>A variant in one of the affected carriers. Figure S1B shows the electropherogram of the unaffected family member, not carrying the variant. Please note that the electropherograms depict the reverse DNA-strand. As such the DNA base change depicted is C>T.


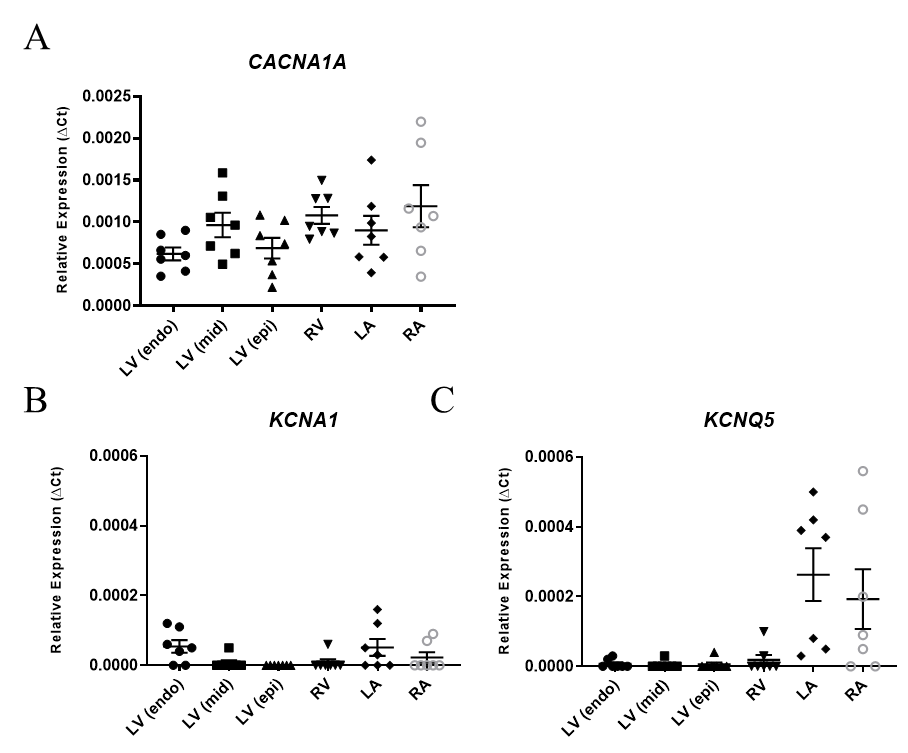


Supplementary Figure S4. Expression of *CACNA1A* in cardiac chambers**.** mRNA level in tissue from the four heart chambers of healthy hearts (n=7). Results are shown as relative expression and error bars represent standard error mean (SEM). The genes *ACTB*, *YWHAZ* and *RPL13A* were used as reference to normalize the data. Data were statistically evaluated using one-way ANOVA followed by a Tukey multiple comparisons test. Abbreviations: LV - left ventricle; endo - endocardium; mid - myocardium; epi - epicardium; RV - right ventricle; LA - left atrium; RA - right atrium.

# Supplementary tables

### Supplementary Table S1. Exome sequencing statistics.

| **ID** | **N. alt** | **N. min** | **N. het** | **Sing.** | **TiTv** | **Mean** | **3d Q*** | **Median*** | **1st Q*** | **%>10X** | **%>20X** |
| --- | --- | --- | --- | --- | --- | --- | --- | --- | --- | --- | --- |
| III-1 | 35331 | 23249 | 21383 | 1753 | 2.78 | 82.36 | 119 | 60 | 21 | 85 | 75.7 |
| III-2 | 34882 | 23251 | 21064 | 1668 | 2.67 | 87.18 | 122 | 68 | 30 | 89.8 | 82 |
| II-4 | 34990 | 22320 | 20887 | 693 | 2.71 | 94.15 | 133 | 72 | 30 | 89.5 | 81.9 |
| II-1 | 35351 | 22591 | 21322 | 639 | 2.71 | 94.87 | 135 | 72 | 29 | 88.6 | 80.7 |
| II-5 | 35562 | 22480 | 21258 | 394 | 2.71 | 95.96 | 136 | 73 | 30 | 89.2 | 81.6 |
| II-6 | 35581 | 21901 | 21409 | 70 | 2.71 | 93.54 | 131 | 72 | 32 | 90.3 | 83 |
| II-2 | 36034 | 23208 | 21869 | 579 | 2.72 | 50.01 | 62 | 36 | 20 | 88.4 | 73.7 |

N = number. Alt = Alternative. Min = minor. Het = Heterozygote. Sing = Singeltons. TiTv = Transition transversion ratio. Q = quartile. X = read depth. *granular.

### Supplementary Table S2. Reported and observed relatedness in Family A

| **ID1** | **ID2** | **IBS0** | **Kinship** | **Observed relation** | **Expected kinship** | **Reported relation** |
| --- | --- | --- | --- | --- | --- | --- |
| III-1 | III-2 | 0.023 | 0.304 | FS | 0.25 | FS |
| III-1 | II-4 | 0.044 | 0.159 | Deg2 | 0.125 | Av |
| III-1 | II-1 | 0.042 | 0.164 | Deg2 | 0.125 | Av |
| III-1 | II-5 | 0.038 | 0.178 | FS | 0.125 | Av |
| III-1 | II-6 | 0.000 | 0.277 | PO | 0.25 | PO |
| III-1 | II-2 | 0.047 | 0.158 | Deg2 | 0.125 | Av |
| III-2 | II-4 | 0.054 | 0.132 | Deg2 | 0.125 | Av |
| III-2 | II-1 | 0.043 | 0.161 | Deg2 | 0.125 | Av |
| III-2 | II-5 | 0.049 | 0.145 | Deg2 | 0.125 | Av |
| III-2 | II-6 | 0.000 | 0.271 | PO | 0.25 | PO |
| III-2 | II-2 | 0.046 | 0.153 | Deg2 | 0.125 | Av |
| II-4 | II-1 | 0.021 | 0.282 | FS | 0.25 | FS |
| II-4 | II-5 | 0.024 | 0.259 | FS | 0.25 | FS |
| II-4 | II-6 | 0.020 | 0.256 | FS | 0.25 | FS |
| II-4 | II-2 | 0.024 | 0.274 | FS | 0.25 | FS |
| II-1 | II-5 | 0.021 | 0.267 | FS | 0.25 | FS |
| II-1 | II-6 | 0.020 | 0.283 | FS | 0.25 | FS |
| II-1 | II-2 | 0.029 | 0.244 | FS | 0.25 | FS |
| II-5 | II-6 | 0.016 | 0.301 | FS | 0.25 | FS |
| II-5 | II-2 | 0.014 | 0.308 | FS | 0.25 | FS |
| II-6 | II-2 | 0.018 | 0.288 | FS | 0.25 | FS |

IBS0= proportion of zero identity by state. Kinship = Kinship coefficient. FS= First sibling. PO = Parent offspring. Av = avuncular.

### Supplementary Table S3. Electrophysiological parameters

|  | **Ca_V_2.1 WT** | **Ca_V_2.1 V1686M** | **p-value** |
| --- | --- | --- | --- |
| Current density (pA/pF) measured at 10 mV | -21.8±2.6  (n=32) | -21.2±1.9  (n=25) | 0.99 |
| V_50_ of activation (mV) | -1.0±0.8  (n=30) | 2.4±0.6  (n=20) | 0.0034 |
| V_50_ of inactivation (mV) | -38.98±2.683  (n=20) | -35.72±1.267  (n=18) | 0.2326 |

# Supplementary References

1. Skarsfeldt MA, Jepps TA, Bomholtz SH, Abildgaard L, Sørensen US, Gregers E, et al. pH-dependent inhibition of K2P3.1 prolongs atrial refractoriness in whole hearts. Pflugers Arch - Eur J Physiol. 2016 Jan 5;1–12.
